# Olfactory Examination in Korsakoff's Syndrome: Implications for Early Diagnosis

**DOI:** 10.5402/2011/506167

**Published:** 2011-10-15

**Authors:** Dawn E. Jones, Marie Rowland, R. Martyn Bracewell

**Affiliations:** ^1^School of Psychology, Bangor University, Brigantia Building, Penrallt Road, Bangor, Gwynedd LL57 2AS, UK; ^2^School of Medical Sciences, Bangor University, Brigantia Building, Penrallt Road, Bangor, Gwynedd LL57 2AS, UK

## Abstract

Whilst olfactory dysfunction has been reported in Korsakoff's Syndrome (KS) patients, the diagnostic implications of this have not been fully explored. KS can be difficult to diagnose because cognitive symptoms are similar to other diagnoses. For instance, patients with Frontal Lobe (FL) Syndrome may present with memory impairments that are similar to KS. Participants were given the Benton Visual Retention Test-Fifth Edition (BVRT-V), to identify working memory dysfunction, and a Brief Smell Identification Test (B-SIT), to evaluate olfactory function. B-SIT scores were found to be significantly lower in the KS group compared to the control and FL groups. In contrast, the error scores on the BVRT-V were significantly higher in both the KS and FL groups compared to the healthy control subjects. Therefore, we suggest that olfactory function may aid in the differential diagnosis of patients presenting with working memory dysfunction.

## 1. Introduction

Olfactory dysfunction has been reported in several neurodegenerative conditions such as Alzheimer's disease (AD) and Parkinson's disease (PD) [1] and may be used as a diagnostic tool [2] in these conditions. However, whilst olfactory dysfunction has been reported in Korsakoff's Syndrome (KS), the possibility of using olfactory dysfunction to aid in a differential diagnosis in patients with working memory dysfunction has not been fully explored. 

KS patients usually first present with Wernicke's encephalopathy (WE), characterized by opthalmoplegia, gait abnormalities, confusion, and some signs of short-term memory loss, before developing KS. WE patients typically respond well to thiamine (vitamin B1), but unfortunately, the signs of WE are often overlooked [3], and without proper thiamine treatment, the WE patient may then develop KS. This development into KS is characterized by severe anterograde amnesia and confabulation. 

Acute KS may also benefit from early thiamine treatment [4]; however KS symptoms may be ascribed to other alcohol-related problems, or the memory impairments (especially working memory) may be confused with frontal lobe (FL) dysfunction [4]. Again the critical period for thiamine treatment may be missed. Given the potential of thiamine treatment, improving the diagnostic pick-up rate for early KS would be a useful clinical goal. 

In this study, we examine the relationship between olfaction and memory in KS patients, FL patients, and healthy volunteers to explore the implications of olfactory testing in the diagnosis of KS. We predicted that both patient groups would perform poorly on the memory test and that KS patients would show a more severe olfactory deficit than FL patients.

## 2. Method

Twelve KS patients, four age-matched FL patients (one left frontoparietal, two right frontotemporal, and one bilateral frontal lesions, due to stroke) and nine healthy age-matched controls were tested. Ethical approval was obtained from the School of Psychology at Bangor University and the North West Wales Research Ethics Committee before this research began. Twelve individuals with a clinical diagnosis of alcoholic Korsakoff's syndrome and a history of Wernicke's encephalopathy were recruited from 4 nursing and private care facilities in the UK. Written and verbal informed consent was obtained from individual patients and their primary caregiver or family member. Medical or nursing care facility admission notes were obtained to confirm diagnosis. The diagnostic guidelines used to verify Wernicke-Korsakoff syndrome were based on consensus guidelines used for inclusion in an ongoing biomedical study investigating the genetic susceptibility of alcohol-related brain damage. The specific guidelines used were

1. Wernicke's encephalopathy—confirmed by medical history of hospitalization with a presentation of confusion and either ataxia or nystagmus;
2. Korsakoff's syndrome—confirmed by a medical history of memory impairment and a past presentation of either confabulation or disorientation, as well as a present indication of short-term memory impairment.

Further selection processes were used to create a homogenous sample of Korsakoff's patients. Only adults under the age of 65 were included. Only individuals with an onset date of 6 months prior to recruitment were included to ensure that the patient group represented those in a chronic stage of the illness. The onset date of Korsakoff's syndrome recorded in their medical or care facility admission notes varied from 6 months to 10 years prior to recruitment. Only individuals who were currently abstinent from alcohol, and had been for at least 6 months, were included. Individuals with coexisting brain injuries, psychological, or neurological diagnoses were not included. There was no indication of spontaneous confabulation in any individual included in the Korsakoff's group. All Korsakoff's patients lived in a care environment where proper nutrition and medical attention were offered, although there was variation in the administration of prescription medication and vitamin supplementation, including thiamine. 

The Benton Visual Retention Test-Fifth Edition (BVRT-V) and the Brief Smell Identification Test (B-SIT). The Brief Smell Identification Test (B-SIT) was administered. A low score indicates poor performance. The Benton Visual Retention Test-Fifth Edition (BVRT-V) was administered. Performance was quantified by the numerical error score calculated relative to the participants IQ and age. A high score indicates a deficit in working memory. Testing time took approximately 15 minutes in total for each participant. Performance was assessed by the number error score calculated relative to the participants' IQ and age. The 12-item B-SIT [5, 6] was used to evaluate olfactory function. Testing time took approximately 15 minutes in total for each participant.

## 3. Results

**Figure 1:**
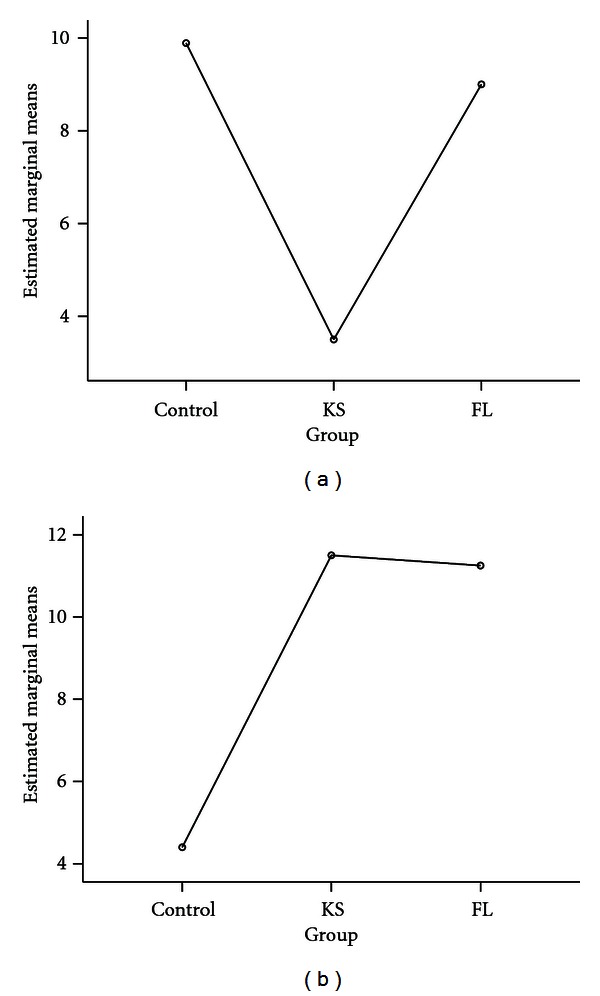
Estimated marginal means of B-SIT scores correct and BVRT-V error scores. (a) Mean difference in number of B-SIT scored correctly between KS, FL, and healthy control group. Olfactory deficits appeared to be absent from the control and FL groups, but present in the KS subjects, as predicted. (b) Mean difference in BVRT-V raw scores between KS, FL, and healthy control group. The number of errors made, relative to age and IQ, were high in both patient groups, with the healthy control participants scoring at normal performance, making few or no errors on the memory assessment.

The B-SIT scores were significantly lower in the KS group than in either the healthy control or FL groups (Figure 1(a)). One-way ANOVA revealed a significant main effect of group (*F*(2,22) = 72.20, *P* < .001). Post hoc multiple comparisons using Tukey's HSD revealed significant differences between the KS and healthy controls groups (mean difference of 6.39, *P* ≤ .001) and also between the KS and FL groups (mean difference of 5.50, *P* ≤ .001). 

In contrast, the KS and FL patients had similar high scores on the BVRT-V, compared to the healthy age-matched control subjects, whereas only the KS patients performed poorly on the B-SIT; therefore the poor performance of KS patients on the B-SIT cannot be attributed to a working memory dysfunction, rather it reflects olfactory dysfunction (Figure 1(b)). One-way ANOVA revealed a main effect of group (*F*(2,10) = 6.00, *P* > .005). Post hoc multiple comparisons using Tukey's HSD revealed significant differences interaction between the healthy control and KS groups (mean difference of 7.10, *P* ≤ .05) and also between the FL and control groups (mean difference of 6.85, *P* ≤ .05).

## 4. Discussion

The present study revealed significant impairment in odour identification in KS patients only. The observed deficits in olfactory function support previous studies [5, 7]. In contrast, FL patients showed normal olfactory function despite evidence of memory impairment similar to KS patients. This supports our suggestion that assessment of olfactory dysfunction may aid in the differential diagnosis of patients presenting with working memory dysfunction.

Chronic alcohol misuse typically results in deficits in memory, planning, organizing, judgment making, and social skills. Since these are difficulties usually related to frontal lobe damage, the presentation of these symptoms in KS or FL patients might be easily confused. The diencephalic pathology seen in KS may be responsible for the selective impairment in olfactory perception observed, which may explain why the loss of perception is characteristic to the disorder [8]. 

Clinical diagnosis of KS is subject to clinical error. When comparing two surveys conducted on the detection rate of KS, it was revealed that standardised tests can often miss the disorder altogether [9]. 

The literature documenting olfactory dysfunction in KS patients is relatively limited and sometimes contradictory [4, 6]. In Hulshoff's et al. (2001) study [10], six FL patients and seven KS patients were tested on odour discrimination. These authors claimed that FL patients were significantly impaired on odour discrimination tasks compared to the KS group, scoring within minimal impairment percentile ranges. However, patients with traumatic FL damage often present with olfactory problems, due to shearing of the olfactory fibres as they pass through the cribriform plate into the underside of the frontal lobes. It is possible that their FL patients may have sustained damage to the olfactory pathways in the inferior part of the frontal lobe, during neurosurgical interventions to remove tumours. In addition, the FL patients' results only reached statistical significance once subjects who had not reached the odour detection criterion were factored out from analysis.

We acknowledge that the present study involved a relatively small number of patients, but the results suggest that assessment of olfactory function may be an effective tool in the differential diagnosis of KS patients from patients with memory dysfunction due only to frontal lobe damage. The nature of KS that often keeps patients in long-term nursing care and the potential for treatment if diagnosed early highlight the importance of an early diagnosis. The B-SIT is an uncomplicated, easily administered assessment tool that is simple for KS and FL patients to complete. The use of the B-SIT could aid in diagnosis and referral for appropriate thiamine treatment. 

Future studies that include a greater number of patients, as well as comparisons to olfactory performance in other neurological disorders, may be useful in further determining the potential value of the B-SIT.
